# CENPA as a Genome Stability–Associated Biomarker in Hepatocellular Carcinoma: Multiomics Analysis and Experimental Validation

**DOI:** 10.1155/humu/9292971

**Published:** 2026-05-25

**Authors:** Haichao Shi, Litao Sun, Pengyan Zhang, Ruizhong Ye, Min Lai, Meixia Du

**Affiliations:** ^1^ Cancer Center, Department of Ultrasound Medicine, Zhejiang Provincial People′s Hospital, Affiliated People′s Hospital, Hangzhou Medical College, Hangzhou, Zhejiang, China, hznu.edu.cn

**Keywords:** CENPA, chromosomal instability, cisplatin sensitivity, genomic instability, hepatocellular carcinoma, multiomics integration

## Abstract

Genomic instability is closely involved in hepatocellular carcinoma (HCC) progression, but biomarkers that reflect genome stability–related tumor biology and functional vulnerability remain incompletely defined. In this study, we performed an integrative multiomics analysis to identify genome stability–associated candidates in HCC and experimentally evaluated the functional relevance of CENPA. By intersecting genome stability–related genes with differentially expressed and survival‐associated genes in TCGA‐LIHC, we identified a candidate gene set enriched in mitotic regulation, DNA maintenance, and cell‐cycle pathways. A LASSO Cox model was then constructed to derive a 10‐gene prognostic signature. Among these genes, CENPA was selected for focused analysis because of its contribution to the model and its established role in centromere identity and chromosome segregation. CENPA was significantly upregulated in HCC tissues, associated with advanced clinicopathological features, and correlated with unfavorable survival outcomes. Copy‐number and cell‐cycle analyses further linked high CENPA expression to proliferative and genome instability–related molecular states. Functional‐state scoring showed strong associations between CENPA expression and cell‐cycle activity, DNA repair, and DNA damage–related programs. In vitro, CENPA knockdown suppressed HCC cell proliferation, clonogenic growth, and migration. Moreover, CENPA depletion increased cisplatin sensitivity, reduced IC50 values, and induced G2/M accumulation in HCC cell line models. Collectively, these findings suggest that CENPA is a genome stability–associated biomarker and functional contributor to malignant phenotypes in HCC. The cisplatin‐related results further indicate a potential association between CENPA‐dependent mitotic regulation and genotoxic stress response, although in vivo and clinical validation are required to determine its translational significance.

## 1. Introduction

Hepatocellular carcinoma (HCC) remains one of the leading causes of cancer‐related mortality worldwide and is characterized by marked molecular heterogeneity and limited therapeutic efficacy in advanced stages [1–3]. Despite advances in surgical approaches, locoregional treatment, and systemic therapies, early detection and precise risk stratification remain major clinical challenges [4, 5]. Genomic instability is a well‐recognized feature of hepatocarcinogenesis and contributes to tumor evolution, intratumoral heterogeneity, and variable therapeutic response [6]. However, although large‐scale sequencing studies have cataloged extensive genetic alterations in HCC, translating these findings into robust and clinically actionable biomarkers remains difficult [7–9]. In particular, biomarkers that reflect genome instability–associated tumor states and can be linked to functional phenotypes require further investigation.

With the rapid development of integrative multiomics approaches, biomarker discovery has gradually shifted from descriptive genomic profiling toward functionally informed molecular interpretation [10, 11]. Rather than focusing exclusively on recurrent coding mutations, recent studies have emphasized the importance of dysregulated genome maintenance pathways, including DNA repair, mitotic control, chromosome segregation, and cell‐cycle regulation [12, 13]. Alterations in these processes may not always result from frequent somatic mutations but can also arise through transcriptional deregulation, copy‐number variation, epigenetic remodeling, or coordinated pathway‐level activation [14, 15]. Therefore, integrating transcriptomic, genomic, and clinical data provides a useful strategy for identifying molecular markers that capture genome instability as a biological state rather than as a single genetic event [16].

Among genome stability regulators, centromere protein A (CENPA) represents a compelling candidate [17]. CENPA is a centromere‐specific histone H3 variant that is indispensable for maintaining centromere identity and ensuring accurate chromosome segregation during mitosis [18]. Dysregulation of CENPA has been implicated in chromosomal missegregation, aneuploidy, and mitotic catastrophe, processes closely linked to oncogenic transformation [19]. Aberrant CENPA expression has been reported in multiple malignancies and is frequently associated with aggressive tumor phenotypes and poor prognosis [20, 21]. Nevertheless, its role in HCC remains incompletely defined, particularly in the context of genome instability and variant‐associated tumor vulnerability [22]. Whether CENPA dysregulation functions as a biomarker reflecting genome instability–associated phenotypes in HCC has not been systematically explored.

In the present study, we applied an integrative multiomics strategy to identify genome stability–related biomarkers in HCC. By intersecting genome stability–associated gene sets with differentially expressed genes (DEGs) and survival‐associated candidates, we constructed a prognostic signature using least absolute shrinkage and selection operator (LASSO) Cox regression. Among the selected genes, CENPA emerged as a stable contributor to risk stratification and a biologically plausible mediator linking chromosomal instability to tumor progression. We therefore performed a comprehensive characterization of CENPA, including expression profiling, clinical correlation, genomic context analysis, and functional validation in HCC models. Through this integrative framework, we aimed to delineate the clinical and biological significance of CENPA and provide new insights into variant‐informed biomarker discovery in HCC.

## 2. Methods

### 2.1. Patient Cohorts and Transcriptomic Data Processing

Transcriptomic and clinical data for HCC were obtained from the Cancer Genome Atlas (TCGA) database (https://portal.gdc.cancer.gov/) [23]. RNA‐seq data from the TCGA‐LIHC cohort generated using the STAR pipeline were downloaded and converted into TPM (transcripts per million) format for downstream analyses [24]. The final dataset included 374 tumor samples and 50 adjacent normal liver tissues, as shown in the descriptive statistics. No additional sample‐level filtering was applied. Gene expression values were transformed using log2(TPM + 1) normalization prior to statistical analyses. Corresponding clinical information, including survival outcomes (overall survival [OS], disease‐specific survival [DSS], progression‐free interval [PFI], and disease‐free interval [DFI]), tumor stage, histological grade, and molecular subtype annotations, was also retrieved for integrative analyses [25].

### 2.2. Identification of Genome Stability–Related Candidate Genes

Genome stability–related genes (GSRGs) were curated from the MSigDB Hallmark collection using the R package msigdbr, integrating genes from HALLMARK_DNA_REPAIR, HALLMARK_G2M_CHECKPOINT, HALLMARK_MITOTIC_SPINDLE, HALLMARK_MYC_TARGETS_V1, and HALLMARK_P53_PATHWAY. Differential expression analysis between HCC and normal liver tissues was conducted using the limma framework with empirical Bayes moderation, and genes with |log2FC| ≥ 1 and *p* < 0.05 were considered DEGs for exploratory candidate screening [26, 27]. OS‐associated genes were identified using univariate Cox proportional hazards regression implemented in the survival package, with *p* < 0.05 regarded as statistically significant for the initial survival‐related screening step [28]. Candidate GSRGs were defined as the intersection of GSRGs, DEGs, and OS‐associated genes and visualized using a Venn diagram. Multiple‐testing correction was not applied during this intersection‐based candidate selection step because the aim was to generate a biologically constrained candidate pool by integrating predefined GSRGs, differential expression, and survival association.

### 2.3. Functional Enrichment Analysis

To explore the biological functions of the candidate genes, functional enrichment analysis was performed using the clusterProfiler package [29, 30]. Gene Ontology (GO) over‐representation analysis was conducted across biological process (BP), cellular component (CC), and molecular function (MF) categories, and KEGG pathway enrichment analysis was performed to identify significantly overrepresented signaling pathways. Enrichment significance was evaluated using a hypergeometric test with false discovery rate (FDR) correction, and terms with adjusted *p* < 0.05 were considered statistically significant. *p* values in GO and KEGG enrichment analyses were adjusted using the Benjamini–Hochberg method. Thus, nominal *p* values were used for exploratory candidate screening, whereas FDR‐adjusted *p* values were used for enrichment analyses.

### 2.4. LASSO Prognostic Model Construction and Evaluation (Methods)

LASSO Cox regression was performed using the R package glmnet to construct a multigene prognostic model [31, 32]. Tenfold cross‐validation was used to determine the optimal penalty parameter (*λ*), and the value corresponding to the minimum cross‐validated partial likelihood deviance (*λ*_min_) was selected to define the final signature; genes with nonzero coefficients at *λ*_min_ were retained. A risk score for each patient was calculated as the weighted sum of expression values multiplied by the corresponding LASSO coefficients. Patients were dichotomized into high‐ and low‐risk groups according to the median risk score. Kaplan–Meier survival curves were generated and compared using the log‐rank test with the survival and survminer packages, and hazard ratios (HRs) with 95% confidence intervals (CIs) were estimated using Cox regression [33]. Time‐dependent receiver operating characteristic (ROC) curves and area under the curve (AUC) values at predefined time points were calculated using the timeROC package [34].

### 2.5. Expression, Diagnostic, and Clinical Correlation Analyses of CENPA

Expression data and clinical annotations for TCGA‐LIHC were obtained from publicly available datasets. Differential expression between tumor and normal tissues was assessed using Wilcoxon′s rank‐sum tests for unpaired samples and paired Wilcoxon′s signed‐rank tests for matched tumor–normal pairs. Diagnostic performance was evaluated using ROC analysis implemented in the pROC package, and the AUC was calculated to quantify discriminative ability [35]. The ROC analysis was performed to evaluate tumor‐versus‐normal tissue discrimination within the TCGA‐LIHC transcriptomic cohort and was not intended to establish clinical diagnostic utility. Associations between CENPA expression and clinicopathological characteristics, including histological grade, pathological stage, and molecular subtype, were evaluated using nonparametric tests (Kruskal–Wallis test followed by Dunn′s post hoc comparison where applicable). Immune subtype classification was based on previously defined TCGA immune classes (C1–C4), and differences in expression among immune subtypes were assessed using Kruskal–Wallis tests. Survival analyses for OS, DSS, DFI, and PFI were conducted using the Kaplan–Meier method with log‐rank tests implemented in the survival and survminer packages. Patients were stratified into high‐ and low‐expression groups according to the median expression of CENPA.

### 2.6. Copy‐Number Alteration, Cell‐Cycle Program, and eQTL–GWAS Locus Visualization

Genome‐wide copy‐number alteration profiles for TCGA‐LIHC were summarized using GISTIC2 outputs [36]. For the expression‐stratified CNV‐burden analysis, samples were divided into CENPA expression quartiles (ExpressionType Q1–Q4), and the Fraction of Genome Altered metrics were summarized separately for FGA and FGG, decomposed into copy‐number–altered, gained, and lost genome components as displayed in the figure. To evaluate the association between CNV and transcription, CENPA expression was compared across discrete CNA states (deep deletion, diploid, gain, and amplification) using a Kruskal–Wallis rank‐sum test, followed by pairwise comparisons where applicable. Cell‐cycle coupling was assessed by comparing CENPA expression across annotated cell‐cycle phases using the Kruskal–Wallis test and by visualizing CENPA expression along the inferred cell‐cycle timeline to depict phase‐dependent dynamics. Regional eQTL–GWAS signal concordance at the CENPA locus was visualized using the locuscompare package, which plots −log10(*P*) for overlapping variants and highlights lead variants [37]. In parallel, stacked regional association plots were generated using gassocplot/gassocplot2, displaying eQTL (ENSG00000115163) and GWAS (ebi‐a‐GCST90092003) association signals across the locus with recombination rate and linkage disequilibrium coloring relative to the lead SNP, as shown.

### 2.7. CancerSEA Functional‐State Scoring and Correlation Analysis

To quantify pathway/state‐level functional programs linked to CENPA, we curated 14 tumor functional‐state gene sets from the CancerSEA resource [38]. Per‐sample functional‐state scores were calculated from the transcriptomic expression matrix using the R package GSVA with the *z*‐score method (i.e., *z*‐score–based gene set scoring), yielding a combined activity score for each state in each sample [39]. The resulting state scores were further standardized using the scale function. Associations between CENPA expression *z*‐scores and standardized functional‐state scores were evaluated using Pearson′s correlation analysis, and correlation coefficients (*R*) and corresponding *p* values were reported and visualized as indicated in the figure.

### 2.8. Cell Lines and Cell Culture

The human normal liver cell line LO2 (RRID: CVCL_6926) and HCC cell lines Hep3B (RRID: CVCL_0326) and Huh7 (RRID: CVCL_0336), as well as the human embryonic kidney cell line HEK293 (RRID: CVCL_0045), were obtained from the American Type Culture Collection (ATCC, Manassas, VA, United States). Cells were maintained in Dulbecco′s Modified Eagle Medium (DMEM; Gibco, Cat. No. 11965092) supplemented with 10% fetal bovine serum (FBS; Gibco, Cat. No. 10099141C) and 1% penicillin–streptomycin solution (Gibco, Cat. No. 15140122). All cultures were incubated at 37°C in a humidified atmosphere containing 5% CO_2_. Cells were routinely passaged at 70%–80% confluence and were confirmed to be free of mycoplasma contamination prior to experimentation.

### 2.9. Stable CENPA Knockdown and Validation

Short hairpin RNA (shRNA) sequences targeting human CENPA and a nontargeting control shRNA (shNC) were cloned into the pLKO.1‐puro lentiviral vector (Addgene, Cat. No. 8453). Recombinant lentiviruses were generated by cotransfecting HEK293 cells (RRID: CVCL_0045) with the shRNA plasmids and packaging plasmids psPAX2 (Addgene, Cat. No. 12260) and pMD2.G (Addgene, Cat. No. 12259) using Lipofectamine 3000 (Invitrogen, Cat. No. L3000015) according to the manufacturer′s protocol. Viral supernatants were collected at 48 and 72 h post‐transfection, filtered through a 0.45 *μ*m membrane, and used to infect Hep3B and Huh7 cells in the presence of 8 *μ*g/mL polybrene (Sigma‐Aldrich, Cat. No. TR‐1003‐G). After 48 h of infection, cells were subjected to puromycin selection (2 *μ*g/mL; Beyotime, Cat. No. ST551) for 7–10 days to establish stable knockdown cell lines. The efficiency of CENPA silencing was confirmed by quantitative real‐time PCR analysis prior to subsequent functional experiments.

### 2.10. Quantitative Real‐Time PCR

Total RNA was extracted from cultured cells using TRIzol Reagent (Invitrogen, Thermo Fisher Scientific, Cat. No. 15596026) according to the manufacturer′s instructions. RNA purity and concentration were evaluated by measuring the absorbance at 260/280 nm using a NanoDrop One spectrophotometer (Thermo Scientific). For reverse transcription, 1 *μ*g of total RNA was converted into cDNA using the PrimeScript RT Reagent Kit with gDNA Eraser (Takara, Cat. No. RR047A) to eliminate potential genomic DNA contamination. The reverse transcription reaction was performed under the following conditions: 37°C for 15 min and 85°C for 5 s. Quantitative PCR was carried out using TB Green Premix Ex Taq II (Takara, Cat. No. RR820A) on a QuantStudio 5 Real‐Time PCR System (Applied Biosystems). Each 20 *μ*L reaction mixture contained 10 *μ*L TB Green Premix, 0.4 *μ*L of forward primer (10 *μ*M), 0.4 *μ*L of reverse primer (10 *μ*M), 2 *μ*L cDNA template, and 7.2 *μ*L nuclease‐free water. The amplification protocol consisted of an initial denaturation at 95°C for 30 s, followed by 40 cycles of 95°C for 5 s and 60°C for 30 s. Melt curve analysis was performed to verify amplification specificity. Relative gene expression levels were calculated using the 2^−*Δ* *Δ*Ct^ method with GAPDH serving as the internal reference gene. All reactions were performed in triplicate, and each experiment was independently repeated at least three times. The primer sequences used in this study were as follows:

GAPDH‐F: GCTCTCTGCTCCTCCTGTTC.

GAPDH‐R: TTCCCGTTCTCAGCCTTGAC.

CENPA‐F: ACTCGTGGTGTGGACTTCAA.

CENPA‐R: CTGGAGAGTCCCCGGTATCA.

### 2.11. Colony Formation Assay

To assess long‐term proliferative potential, Hep3B and Huh7 cells were seeded into six‐well plates at a density of 800–1000 cells per well and cultured under standard conditions. For combination experiments, cells were exposed to cisplatin at a final concentration of 1 *μ*M (Sigma‐Aldrich, Cat. No. P4394) 24 h after seeding. Drug treatment was maintained for 48 h, after which the medium was replaced with fresh complete medium, and cells were allowed to grow for an additional 10–14 days. When colonies in the control wells reached approximately 1–2 mm in diameter, cells were washed with phosphate‐buffered saline (PBS), fixed with 4% paraformaldehyde (Biosharp, Cat. No. BL539A) for 20 min, and stained with 0.1% crystal violet solution (Solarbio, Cat. No. G1062) for 20 min at room temperature. Plates were rinsed thoroughly and air‐dried before counting. Colonies consisting of more than 50 cells were scored as positive colonies. Experiments were performed in triplicate and independently repeated at least three times.

### 2.12. Cell Viability Assay

Cell proliferation was evaluated using the Cell Counting Kit‐8 (CCK‐8; Dojindo, Cat. No. CK04). Briefly, Hep3B and Huh7 cells were seeded into 96‐well plates at a density of 3 × 10^3^ cells per well in 100 *μ*L complete medium and allowed to adhere overnight. At the indicated time points, 10 *μ*L of CCK‐8 reagent was added to each well, followed by incubation at 37°C for 1–2 h. The absorbance was measured at 450 nm using a microplate reader (BioTek Synergy HTX, Agilent Technologies). Background absorbance from medium‐only wells was subtracted prior to analysis. For cisplatin sensitivity assays, cells were treated with increasing concentrations of cisplatin (Sigma‐Aldrich, Cat. No. P4394) for 48 h before CCK‐8 detection. Dose–response curves and IC50 values were calculated using nonlinear regression analysis in GraphPad Prism 9 software. All experiments were performed in triplicate wells and repeated at least three times independently.

### 2.13. Wound Healing Migration Assay

Cell migratory capacity was evaluated using a wound healing assay. Hep3B cells were seeded into six‐well plates and cultured until reaching approximately 90% confluence. A linear scratch was generated across the cell monolayer using a sterile 200 *μ*L pipette tip. Detached cells were gently removed by washing twice with PBS. Cells were then cultured in serum‐reduced medium (DMEM containing 1% FBS) to minimize the influence of cell proliferation. Images of the wound area were captured at 0 and 48 h using an inverted microscope (Olympus IX73). Migration rate was calculated as the percentage of wound closure relative to the initial scratch width using ImageJ software (NIH). Each experiment was performed in triplicate and independently repeated at least three times.

### 2.14. Cell‐Cycle Analysis

Cell‐cycle distribution was analyzed by flow cytometry using propidium iodide (PI) staining. Hep3B and Huh7 cells were harvested, washed twice with cold PBS, and fixed in 70% ethanol at 4°C overnight. After fixation, cells were centrifuged and resuspended in staining buffer containing 50 *μ*g/mL PI (Beyotime, Cat. No. C1052) and 100 *μ*g/mL RNase A (Beyotime, Cat. No. ST579). Samples were incubated at 37°C for 30 min in the dark to allow complete RNA digestion and DNA staining. Cell‐cycle profiles were acquired using a BD FACSCanto II flow cytometer (BD Biosciences) and analyzed with FlowJo software (Version 10). The proportions of cells in G0/G1, S, and G2/M phases were determined using the Watson pragmatic model. All experiments were independently performed at least three times.

### 2.15. Statistical Analysis

All statistical analyses were performed using R software (Version 4.1.1) and GraphPad Prism 9. Continuous variables were presented as mean ± standard deviation (SD) unless otherwise specified. Differences between the two groups were evaluated using the Wilcoxon rank‐sum test or Student′s *t*‐test, depending on data distribution. Comparisons among multiple groups were conducted using the Kruskal–Wallis test followed by Dunn′s post hoc test. Survival analyses were performed using the Kaplan–Meier method with log‐rank tests to compare differences between groups. HRs and 95% CIs were calculated using Cox proportional hazards regression models. Time‐dependent ROC curves were used to evaluate predictive performance. Correlation analyses were conducted using Pearson′s or Spearman′s correlation coefficients as appropriate. For exploratory candidate gene screening, nominal *p* < 0.05 was used unless otherwise stated. For GO and KEGG enrichment analyses, adjusted *p* < 0.05 after FDR correction was considered statistically significant. All statistical tests were two‐sided, and a *p* value < 0.05 was considered statistically significant unless otherwise stated.

## 3. Result

### 3.1. Screening of Genome Stability–Related Candidate Genes in HCC

**Figure 1:**
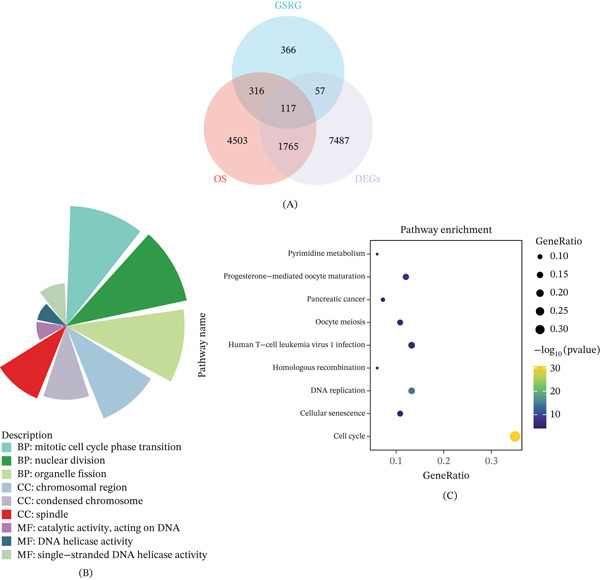
Identification and functional annotation of genome stability–related candidate genes in HCC. (A) Venn diagram showing the overlap among genome stability–related genes (GSRGs), differentially expressed genes (DEGs), and overall survival (OS)–associated genes, resulting in 117 shared candidates. (B) Gene Ontology (GO) enrichment analysis of the overlapping genes, including biological process (BP), cellular component (CC), and molecular function (MF) categories. Enriched terms are primarily associated with mitotic progression and chromosomal regulation. (C) KEGG pathway enrichment analysis of the candidate genes, highlighting significant enrichment in cell‐cycle regulation and DNA replication–related pathways.

To identify genome stability–related candidate genes in HCC, we integrated differential expression, survival analysis, and curated GSRG sets. DEGs were defined as |log2FC| ≥ 1 with *p* < 0.05, and OS‐associated genes were identified using univariate Cox regression with *p* < 0.05. As shown in Figure 1A, intersecting GSRGs, DEGs, and OS‐related genes yielded 117 overlapping genes. Functional enrichment analysis revealed that these candidates were predominantly involved in mitotic regulation and genome maintenance. GO analysis indicated significant enrichment in BPs related to mitotic cell‐cycle phase transition, nuclear division, and organelle fission, as well as CCs, including chromosomal region, condensed chromosome, and spindle. MF enrichment highlighted activities associated with catalytic activity acting on DNA, DNA helicase activity, and single‐stranded DNA helicase activity (Figure 1B). Consistently, KEGG pathway analysis demonstrated significant enrichment in cell cycle, cellular senescence, DNA replication, and homologous recombination, along with additional pathways such as human T‐cell leukemia virus 1 infection, oocyte meiosis, pancreatic cancer, progesterone‐mediated oocyte maturation, and pyrimidine metabolism (Figure 1C). Collectively, these findings indicate that the identified 117 genes capture key regulatory programs associated with genome stability and proliferative control in HCC.

### 3.2. Construction and Performance Evaluation of the LASSO‐Based Prognostic Signature

**Figure 2:**
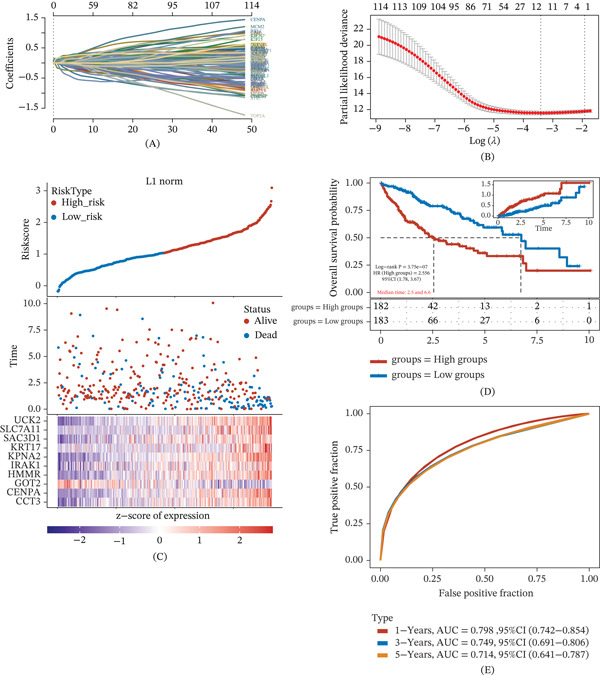
Development and evaluation of a LASSO‐derived genome stability–related prognostic signature. (A) LASSO coefficient profiles of candidate genes across different values of log (*λ*). (B) Tenfold cross‐validation curve for selecting the optimal penalty parameter; the minimum criterion identified *λ*_min_ = 0.0331. (C) Risk score distribution, survival time/status, and expression heat map of the 10‐gene signature in patients ranked by increasing risk score. (D) Kaplan–Meier overall survival curves for high‐ and low‐risk groups stratified by the median risk score (log‐rank *p* = 3.75 × 10^−7^; HR = 2.556; 95% CI, 1.78–3.67; median survival: 2.5 vs. 6.6 years). (E) Time‐dependent ROC curves showing prognostic accuracy at 1, 3, and 5 years (AUC: 0.789, 0.749, and 0.714, respectively).

Using the model genes, we constructed a prognostic signature via LASSO Cox regression with 10‐fold cross‐validation. The coefficient trajectories and cross‐validated partial likelihood deviance are shown in Figure 2A,B, and the optimal penalty parameter was selected at *λ*_min_ = 0.0331, yielding a 10‐gene model consisting of CCT3, CENPA, GOT2, HMMR, IRAK1, KPNA2, KRT17, SAC3D1, SLC7A11, and UCK2. The risk score was calculated as follows: Risk score = (0.02 × CCT3) + (0.0042 × CENPA) − (0.1463 × GOT2) + (0.125 × HMMR) + (0.0493 × IRAK1) + (0.0933 × KPNA2) + (0.0869 × KRT17) + (0.0542 × SAC3D1) + (0.133 × SLC7A11) + (0.1561 × UCK2). Patients were stratified into high‐ and low‐risk groups based on the median risk score, and the distribution of risk scores, survival time/status, and expression pattern of the 10 genes are summarized in Figure 2C. Kaplan–Meier analysis demonstrated significantly poorer OS in the high‐risk group compared with the low‐risk group (log‐rank *p* = 3.75 × 10^−7^; HR = 2.556; 95% CI, 1.78–3.67), with median survival times of 2.5 years (high‐risk) versus 6.6 years (low‐risk) (Figure 2D). Time‐dependent ROC analysis further supported the prognostic performance of the signature, with AUCs of 0.789 (95% CI, 0.742–0.854) at 1 year, 0.749 (95% CI, 0.691–0.806) at 3 years, and 0.714 (95% CI, 0.641–0.787) at 5 years (Figure 2E). These results indicate that the genome stability–related signature showed prognostic discrimination within the TCGA‐LIHC cohort and provided a basis for prioritizing individual model genes for downstream biological analysis.

### 3.3. Multidimensional Characterization of CENPA in HCC

To gain deeper biological insight into the prognostic signature, we next interrogated individual genes retained in the LASSO model. Among these genes, CENPA, a core regulator of centromere integrity and mitotic fidelity, was prioritized for downstream analyses because it contributed to the prognostic model, showed clear tumor‐associated upregulation, and had a direct biological relationship with centromere identity, chromosome segregation, and genome stability. Compared with other model genes, this mechanistic relevance made CENPA particularly suitable for testing the genome stability–centered hypothesis of this study. We therefore conducted a comprehensive multidimensional characterization of CENPA in HCC.

**Figure 3:**
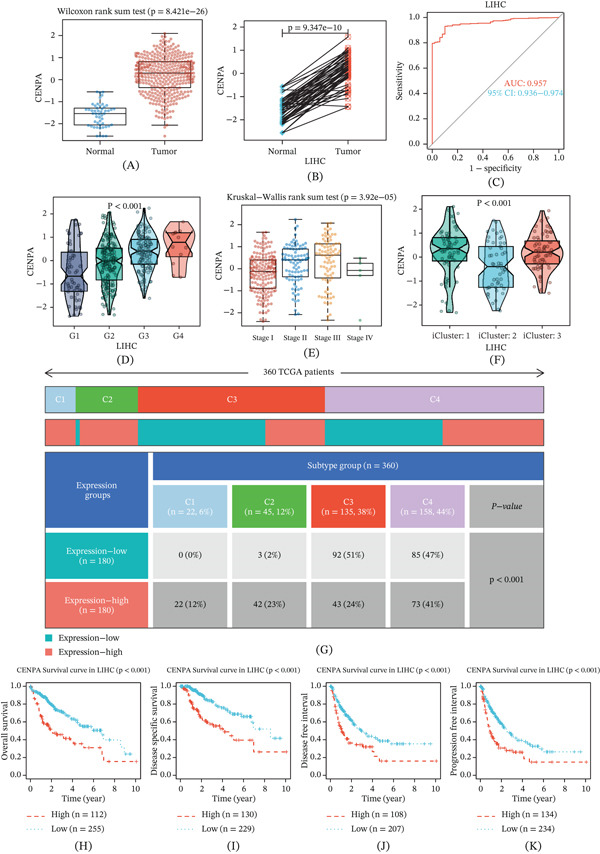
Multidimensional analysis of CENPA in hepatocellular carcinoma. (A) Differential expression of CENPA between tumor and normal tissues in TCGA‐LIHC (unpaired analysis). (B) Paired comparison of CENPA expression between matched tumor and adjacent normal tissues. (C) Receiver operating characteristic (ROC) curve evaluating the diagnostic performance of CENPA (AUC = 0.957). (D) Association between CENPA expression and histological grade (G1–G4). (E) Correlation between CENPA expression and pathological stage (Stages I–IV). (F) Differences in CENPA expression across TCGA molecular subtypes. (G) Distribution of CENPA expression across immune subtypes (C1–C4). (H–K) Kaplan–Meier survival analyses of overall survival (OS), disease‐specific survival (DSS), disease‐free interval (DFI), and progression‐free interval (PFI) stratified by CENPA expression.

Expression analysis in TCGA‐LIHC demonstrated that CENPA was significantly upregulated in tumor tissues compared with normal liver tissues in both unpaired and paired comparisons (Figure 3A,B). ROC analysis further revealed strong diagnostic performance, with an AUC of 0.957, indicating high accuracy in distinguishing tumor from normal samples (Figure 3C). We next assessed the association between CENPA expression and clinicopathological features. Elevated CENPA expression was significantly associated with higher histological grade (G1–G4; *p* < 0.001) and advanced pathological stage (Stages I–IV; *p* = 3.92 × 10^−5^) (Figure 3D,E). In addition, CENPA expression differed significantly across TCGA‐defined molecular subtypes (*p* < 0.001), suggesting subtype‐specific biological heterogeneity (Figure 3F). Immune subtype analysis revealed significant variation in CENPA expression among immune classes (C1–C4; *p* < 0.001), highlighting a potential link between CENPA and the tumor immune contexture (Figure 3G). Finally, survival analyses consistently supported the prognostic relevance of CENPA. Kaplan–Meier curves showed that high CENPA expression was associated with significantly worse OS, DSS, DFI, and PFI compared with low‐expression patients (Figure 3H–K). Collectively, these findings support CENPA as a highly expressed and clinically relevant candidate gene in HCC, associated with tumor aggressiveness, molecular/immune heterogeneity, and adverse outcomes, thereby supporting its prioritization for subsequent multiomics characterization and experimental validation.

### 3.4. Copy‐Number Context, Cell‐Cycle Coupling, Genetic Colocalization, and Functional‐State Correlations of CENPA in HCC

We next investigated whether genomic alterations and functional programs could explain CENPA dysregulation in HCC. GISTIC2 profiling in TCGA‐LIHC (*n* = 370) showed broad arm‐/focal‐level copy‐number changes across the genome, providing a CNV background for downstream CENPA‐centered analyses (Figure 4A). To further link CENPA‐defined expression strata with genome‐wide CNV burden, samples were grouped into ExpressionType Q1–Q4, and the fraction of the genome altered for FGA and FGG was compared across quartiles, with contributions from copy‐number–altered, gained, and lost genome summarized within each stratum (Figure 4B). Consistent with a copy‐number–expression relationship, CENPA expression differed significantly across CNA states (deep deletion, diploid, gain, and amplification; *p* < 0.001), with higher expression observed in gain/amplification categories (Figure 4C). Cell‐cycle analyses further supported proliferation coupling: CENPA expression varied significantly across phases (Kruskal–Wallis rank‐sum test *p* < 0.001), showing the highest distribution in proliferative states (Figure 4D), and exhibited a clear phase‐dependent trajectory along inferred cell‐cycle time (Figure 4E). To explore whether genetic regulation of CENPA expression shares a causal variant with HCC‐related GWAS signals, we visualized the eQTL–GWAS locus using locuscompare and stacked association plots. This analysis was performed as a locus‐level visualization rather than a formal statistical colocalization test. The locuscompare plot showed limited concordance between eQTL and GWAS association patterns, with the eQTL lead SNP labeled as rs142326416 (Figure 4F). In the stacked regional association plots, the eQTL signal for ENSG00000115163 (CENPA) and the GWAS signal for ebi‐a‐GCST90092003 peaked at different lead variants (rs142326416 vs. rs12487466), providing no obvious visual evidence for a shared lead signal at this locus (Figure 4G). Therefore, these results should be interpreted as a negative or inconclusive locus‐level visualization rather than definitive evidence against any regulatory relationship.

Figure 4Copy‐number context, cell‐cycle coupling, genetic colocalization visualization, and CancerSEA functional‐state correlations of CENPA in HCC. (A) Genome‐wide GISTIC2 copy‐number score profile in TCGA‐LIHC (*n* = 370). (B) Fractions of the genome altered for FGA and FGG across ExpressionType Q1–Q4, with copy‐number–altered, gained, and lost genome components displayed. (C) CENPA expression across CNA states (deep deletion, diploid, gain, and amplification; *p* < 0.001). (D) CENPA expression distributions across cell‐cycle phases (Kruskal–Wallis rank‐sum test *p* < 0.001). (E) CENPA expression trend along inferred cell‐cycle time with phase annotations. (F) Locuscompare visualization comparing eQTL and GWAS signals at the CENPA locus; eQTL lead variant labeled (rs142326416). (G) Stacked regional association plots for the eQTL (ENSG00000115163) and GWAS (ebi‐a‐GCST90092003) signals; lead variants labeled (rs142326416 vs. rs12487466). (H) CancerSEA functional‐state correlations with CENPA expression, reporting Pearson′s R and *p* values for 14 states.

**Figure:**
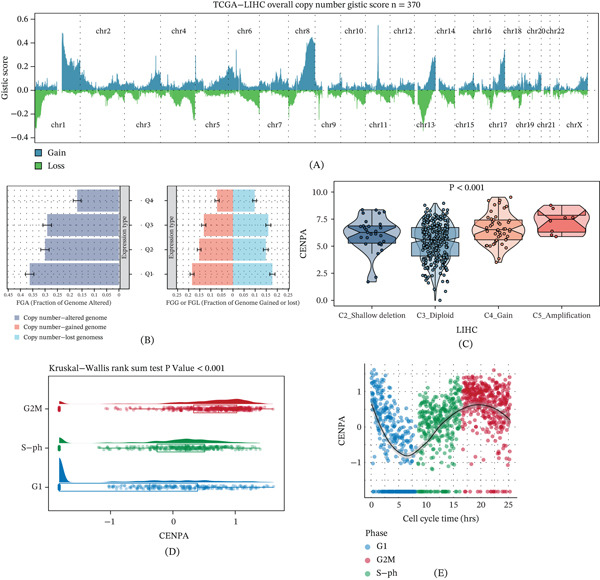
(A)

**Figure:**
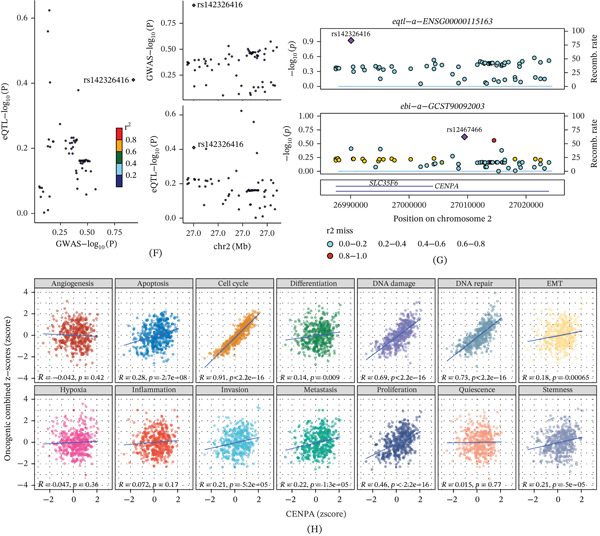
(B)

Finally, CancerSEA‐based functional‐state analysis revealed that CENPA expression was most strongly correlated with cell‐cycle activity (*R* = 0.91, *p* < 2.2 × 10^−16^) and also showed robust positive correlations with DNA repair (*R* = 0.73, *p* < 2.2 × 10^−16^), DNA damage (*R* = 0.69, *p* < 2.2 × 10^−16^), and proliferation (*R* = 0.46, *p* < 2.2 × 10^−16^) (Figure 4H). Moderate but significant associations were observed for apoptosis (*R* = 0.28, *p* = 2.7 × 10^−8^), invasion (*R* = 0.21, *p* = 5.2 × 10^−5^), metastasis (*R* = 0.22, *p* = 1.3 × 10^−5^), stemness (*R* = 0.21, *p* = 5 × 10^−5^), EMT (*R* = 0.18, *p* = 6.5 × 10^−4^), and differentiation (*R* = 0.14, *p* = 0.009), whereas angiogenesis, hypoxia, inflammation, and quiescence showed weak or nonsignificant correlations (Figure 4H). Together, these results support that CENPA upregulation in HCC is associated with CNV state and proliferative programs, while the current locus‐level evidence does not indicate an obvious shared lead variant between the tested eQTL and GWAS signals.

### 3.5. CENPA Promotes Malignant Phenotypes in HCC Cells

**Figure 5:**
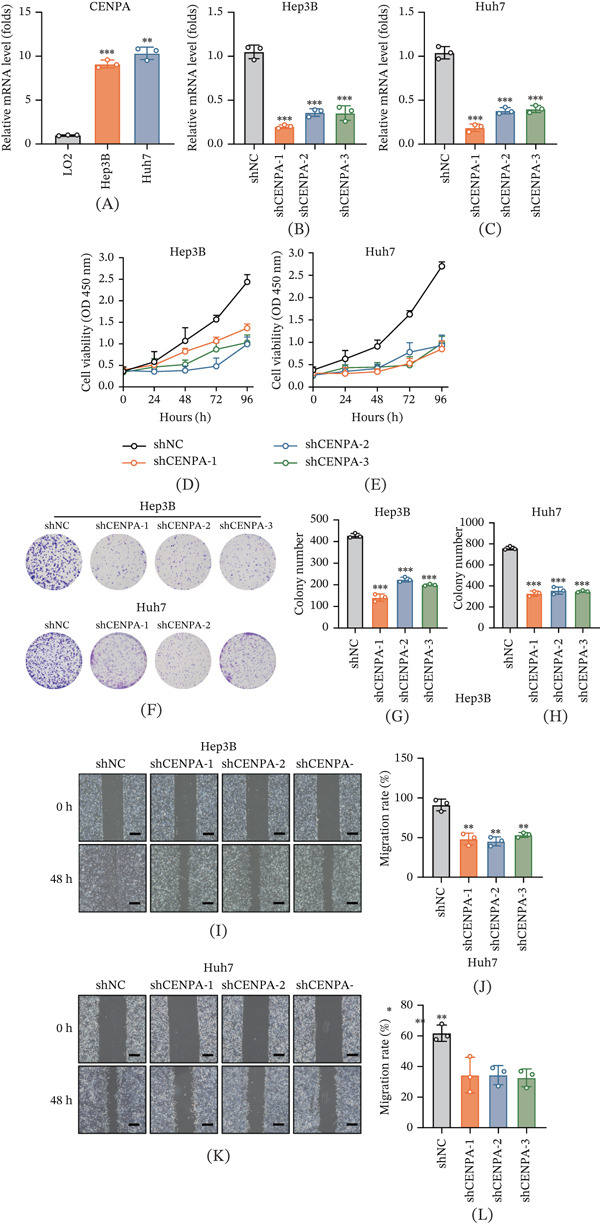
CENPA knockdown suppresses malignant phenotypes in hepatocellular carcinoma cells. (A) Relative CENPA mRNA expression measured by quantitative real‐time PCR (qRT‐PCR) in the normal hepatic cell line LO2 and HCC cell lines Hep3B and Huh7. Expression was normalized to GAPDH and presented relative to LO2. Establishment and validation of stable CENPA knockdown in (B) Hep3B and (C) Huh7 cells. Cells were transduced with a control shRNA (shNC) or three independent CENPA‐targeting shRNAs (shCENPA‐1, shCENPA‐2, and shCENPA‐3), and CENPA mRNA levels were quantified by qRT‐PCR with GAPDH normalization and presented relative to shNC. CCK‐8 assays showing cell viability/proliferation curves for (D) Hep3B and (E) Huh7 cells transduced with shNC or CENPA‐targeting shRNAs at the indicated time points (0–96 h). Absorbance was measured at 450 nm. (F) Representative images of colony formation assays performed in Hep3B and Huh7 cells following stable transduction with shNC or CENPA‐targeting shRNAs. Quantification of colony numbers in (G) Hep3B and (H) Huh7 cells. Colonies were counted according to the predefined criterion described in the Methods section. Wound healing assay in Hep3B cells: (I) representative images at 0 and 48 h after scratching and (J) quantification of migration rate expressed as percentage wound closure. Wound healing assay in Huh7 cells: (K) representative images at 0 and 48 h after scratching and (L) quantification of migration rate expressed as percentage wound closure. Data are presented as mean ± SD from three independent experiments. Statistical significance was determined as described in the Methods section.  ^∗^ *p* < 0.05,  ^∗∗^ *p* < 0.01, and  ^∗∗∗^ *p* < 0.001 versus shNC.

To experimentally validate the protumorigenic role of CENPA in HCC, we first quantified its basal expression in normal and malignant liver cell lines. Quantitative real‐time PCR showed that CENPA mRNA levels were significantly higher in the HCC cell lines Hep3B and Huh7 than in the normal hepatic cell line LO2 (Figure 5A), indicating that CENPA is transcriptionally upregulated in HCC cells in vitro. We next established stable loss‐of‐function models to evaluate the functional contribution of CENPA. Hep3B and Huh7 cells were transduced with a control shRNA (shNC) or three independent CENPA‐targeting shRNAs (shCENPA‐1, shCENPA‐2, and shCENPA‐3), followed by puromycin selection to obtain stable knockdown cell lines. qRT‐PCR validation confirmed robust and consistent suppression of CENPA expression across the three shRNA constructs in both Hep3B and Huh7 cells compared with the shNC group (Figure 5B,C), supporting the reliability of these models for downstream phenotypic assays.

Cell proliferation was then assessed using the CCK‐8 assay across a 96‐h time course. In both Hep3B and Huh7 cells, CENPA knockdown led to a sustained reduction in cell viability, with the divergence from the control group becoming increasingly apparent over time (Figure 5D,E). This time‐dependent inhibitory effect observed across multiple independent shRNAs indicates that CENPA contributes to proliferative capacity in HCC cells rather than representing an off‐target artifact of a single construct. To further evaluate long‐term clonogenic potential, colony formation assays were performed. Representative crystal violet–stained plates showed visibly fewer colonies in CENPA‐silenced groups relative to shNC controls for both Hep3B and Huh7 cells (Figure 5F). Quantification confirmed that CENPA depletion significantly reduced colony numbers, indicating impaired clonogenic survival and long‐term proliferative fitness following CENPA silencing (Figure 5G,H).

Finally, we investigated whether CENPA influences cell migratory behavior using wound healing assays. Representative images captured at 0 and 48 h demonstrated that the wound area closed substantially in shNC cells but remained markedly wider in CENPA knockdown cells (Figure 5I–K). Quantitative analysis of wound closure further verified that CENPA knockdown significantly decreased migration rates in both Hep3B and Huh7 cells compared with controls (Figure 5J,L). Together, these functional assays consistently show that CENPA contributes to multiple malignant phenotypes of HCC cells, including proliferation, clonogenicity, and migration.

### 3.6. CENPA Depletion Increases Cisplatin Sensitivity and Induces G2/M Arrest in HCC Cells

**Figure 6:**
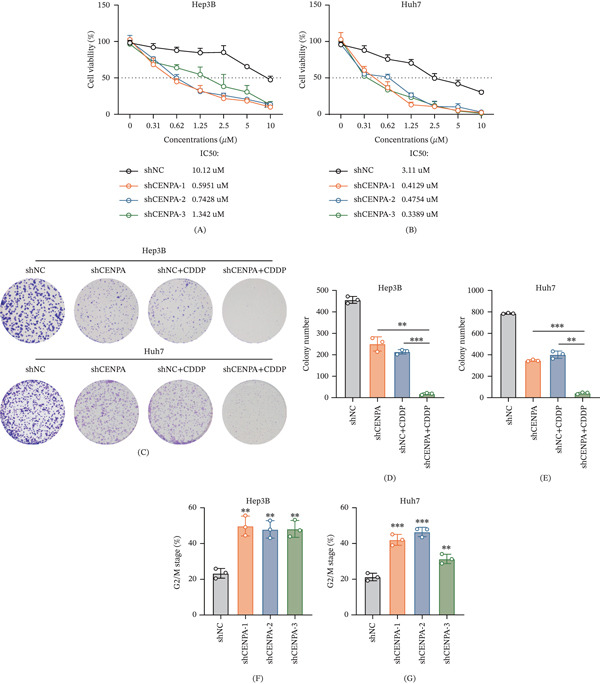
CENPA depletion increases cisplatin sensitivity and induces G2/M accumulation in hepatocellular carcinoma cell models. Dose–response curves of (A) Hep3B and (B) Huh7 cells transduced with shNC or CENPA‐targeting shRNAs following treatment with increasing concentrations of cisplatin (CDDP). Cell viability was assessed by CCK‐8 assay, and IC50 values were calculated using nonlinear regression. (C) Representative colony formation images of Hep3B and Huh7 cells treated with shNC, shCENPA, shNC + CDDP, or shCENPA + CDDP. Quantification of colony numbers in (D) Hep3B and (E) Huh7 cells under the indicated conditions, showing enhanced clonogenic suppression with combined CENPA knockdown and cisplatin treatment. Flow cytometric cell‐cycle analysis showing the percentage of cells in the G2/M phase in (F) Hep3B and (G) Huh7 cells after CENPA knockdown. Data are presented as mean ± SD from three independent experiments. Statistical analysis was performed as described in the Methods section.  ^∗∗^ *p* < 0.01, and  ^∗∗∗^ *p* < 0.001.

Given the central role of CENPA in chromosome segregation and genome stability, we next examined whether its depletion confers vulnerability to DNA‐damaging stress by assessing cellular response to cisplatin. First, dose–response CCK‐8 assays demonstrated that CENPA knockdown markedly enhanced cisplatin sensitivity in both HCC cell lines (Figure 6A,B). In Hep3B cells, CENPA silencing shifted the viability curve downward and substantially reduced the IC50 from 10.12 *μ*M in shNC cells to 0.5951, 0.7428, and 1.342 *μ*M in shCENPA‐1/2/3 cells, respectively (Figure 6A). Similarly, in Huh7 cells, CENPA depletion reduced the IC50 from 3.11 *μ*M in shNC cells to 0.4129, 0.4754, and 0.3389 *μ*M in the three shCENPA groups (Figure 6B), indicating a consistent increase in cisplatin susceptibility across independent shRNAs. To further test whether this increased drug sensitivity translates into long‐term growth suppression, clonogenic assays were performed under four conditions: shNC, shCENPA, shNC + cisplatin (CDDP), and shCENPA + CDDP (Figure 6C–E). Representative colony formation images showed that either CENPA knockdown or cisplatin alone reduced colony formation compared with shNC controls, whereas the combination of CENPA silencing and cisplatin led to the most pronounced inhibition of clonogenic survival in both Hep3B and Huh7 cells (Figure 6C). Quantification confirmed that colony numbers were significantly lower in the combined treatment group compared with either intervention alone in Hep3B (Figure 6D) and Huh7 cells (Figure 6E), supporting a cooperative suppressive effect on long‐term proliferative capacity.

Finally, given that CENPA is required for faithful mitotic progression, we assessed whether its depletion alters cell‐cycle distribution. Flow cytometric analysis revealed a significant accumulation of cells in the G2/M phase following CENPA knockdown in both Hep3B and Huh7 cells (Figure 6F,G), indicating G2/M arrest and impaired cell‐cycle progression. Collectively, these data demonstrate that CENPA depletion increases cisplatin sensitivity, compromises clonogenic survival under genotoxic stress, and induces G2/M phase accumulation in HCC cell line models. These findings support a potential association between CENPA‐dependent mitotic regulation and cisplatin response, while broader therapeutic implications require further mechanistic, in vivo, and clinical validation.

## 4. Discussion

Genomic instability is a central biological feature of HCC, shaping tumor evolution, intratumoral heterogeneity, and therapeutic response [6, 40]. In addition to recurrent somatic mutations, instability‐related phenotypes may also arise from deregulated genome maintenance programs, including mitotic progression, centromere integrity, chromosome segregation, and DNA damage response [41]. Based on this rationale, the present study used a genome stability–anchored screening strategy rather than an unrestricted transcriptome‐wide marker search. By integrating GSRG sets with differential expression and survival association in TCGA‐LIHC, we identified a candidate gene set enriched in cell‐cycle regulation, DNA replication, homologous recombination, and other genome maintenance pathways. This result supports the biological relevance of the screening strategy and suggests that the selected genes reflect proliferative and genome stability–related molecular states in HCC.

Within this framework, CENPA was selected for focused investigation because it combined prognostic relevance with a direct biological relationship to chromosome segregation. CENPA encodes a centromere‐specific histone H3 variant required for centromere identity and accurate kinetochore assembly [42]. Dysregulated CENPA expression has been associated with chromosomal missegregation, aneuploidy, and aggressive behavior in multiple cancer types [19, 43]. In our analyses, CENPA was markedly upregulated in HCC tissues and was associated with higher histological grade, advanced pathological stage, molecular subtype differences, immune subtype variation, and poorer survival outcomes. These observations suggest that CENPA is not only a component of the LASSO‐derived prognostic signature but also a biologically plausible candidate marker linked to aggressive HCC phenotypes. However, these findings should be interpreted as retrospective cohort‐based associations rather than evidence that CENPA has immediate clinical diagnostic or prognostic utility.

Beyond expression–outcome associations, the multiomics analyses further placed CENPA within a proliferative and genome instability–associated molecular context. Copy‐number alteration is a major substrate of genomic instability, and genome‐wide CNA burden has been associated with tumor progression and immune microenvironment remodeling in multiple cancers, including HCC [44]. In our analyses, stratifying samples by CENPA expression linked high expression with increased CNA‐related features, and comparing expression across discrete CNA states further supported a gene dosage–compatible pattern. While this does not establish causality, it is consistent with a model in which CENPA‐high tumors represent a chromosomally unstable state enriched for copy‐number remodeling and proliferative advantage. In parallel, functional‐state scoring and pathway‐level inference indicated that higher CENPA expression aligns with programs related to cell cycle and genome maintenance, reinforcing the interpretation that CENPA is embedded within a broader instability/proliferation axis rather than acting as an isolated marker.

The experimental validation strengthens this interpretation by directly connecting CENPA to malignant phenotypes in HCC cell models. Silencing CENPA suppressed proliferative capacity (CCK‐8), reduced long‐term clonogenic survival, and impaired migratory ability, which is consistent with the expected dependence of rapidly dividing tumor cells on faithful chromosome segregation. Notably, these functional effects were observed using multiple independent shRNAs across two HCC cell lines, improving confidence that the phenotype reflects on‐target CENPA depletion rather than a sequence‐specific artifact. Together, the data support an oncogenic dependency on CENPA in HCC cells, aligning with prior evidence that cancer cells often exploit or become dependent on mitotic regulators to sustain high proliferation under genomic stress [45].

A particularly translational aspect of this study is the observation that CENPA depletion increases sensitivity to cisplatin and is accompanied by G2/M accumulation [46]. DNA‐damaging agents such as cisplatin can preferentially impact tumor cells with defective damage tolerance or compromised mitotic fidelity, and chromosomal instability has long been discussed as a double‐edged sword—promoting evolution and resistance while also creating therapeutic liabilities [40, 47]. The marked reduction in cisplatin IC50 following CENPA knockdown, together with the enhanced suppression in clonogenic assays under combined perturbation, suggests that CENPA contributes to genotoxic stress tolerance in HCC cells. The accompanying G2/M accumulation is biologically coherent: Perturbing centromere function and mitotic progression can activate checkpoint control and exacerbate mitotic stress, thereby limiting the capacity to complete cell division under DNA damage. While the current work does not dissect downstream DNA damage signaling or repair pathway activity, the phenotype is compatible with a model where CENPA supports mitotic robustness and enables survival under genotoxic insult.

Several limitations should be acknowledged. First, the computational analyses were mainly derived from TCGA‐LIHC, and the prognostic model was not externally validated in an independent HCC cohort. Therefore, the generalizability of the survival‐related findings remains to be confirmed. Second, although CENPA showed strong tumor‐versus‐normal discrimination, this ROC analysis was based on a retrospective tissue transcriptomic cohort and should not be interpreted as evidence of immediate clinical diagnostic applicability. Third, the observed associations between CENPA expression, CNA features, cell‐cycle programs, and functional‐state scores are correlative and may be influenced by tumor purity, proliferation rate, and other confounding factors. Fourth, although the in vitro results support the functional relevance of CENPA, no xenograft or other in vivo model was performed in the present study. Finally, because direct assays of DNA damage accumulation, replication stress, or chromosomal segregation defects were not included, the mechanistic connection between CENPA depletion and genome instability–associated vulnerability remains incompletely defined. Future studies incorporating in vivo models, rescue experiments, and dedicated genome instability assays will be needed to further clarify the biological and translational significance of CENPA in HCC.

## 5. Conclusion

In summary, this study identifies CENPA as a genome stability–associated biomarker candidate in HCC through integrative multiomics analysis and functional validation. CENPA is upregulated in HCC, associated with adverse clinical features and survival outcomes, and linked to proliferative and genome maintenance–related programs. In vitro, CENPA knockdown suppresses malignant phenotypes, increases cisplatin sensitivity, and induces G2/M accumulation in HCC cell models. These findings support the biological relevance of CENPA in HCC, while further mechanistic, in vivo, and clinical validation are needed to determine its translational significance.

## Author Contributions

Haichao Shi and Meixia Du conceived and designed the study. Haichao Shi performed the majority of experiments and bioinformatics analyses and drafted the manuscript. Litao Sun and Pengyan Zhang contributed to experimental validation and data analysis. Ruizhong Ye provided technical support and resources. Min Lai and Meixia Du supervised the project and revised the manuscript. All study design, data analysis, interpretation, and scientific conclusions were developed by the authors.

## Funding

This work was supported by the Zhejiang Medicine Scientific and Technology Project (Grant No. 2022PY003).

## Disclosure

All authors approved the final version. The authors take full responsibility for the integrity and originality of the work.

## Conflicts of Interest

The authors declare no conflicts of interest.

## Data Availability

All datasets analyzed in this study are publicly available. Transcriptomic and clinical data were obtained from the Cancer Genome Atlas (TCGA‐LIHC) via the Genomic Data Commons portal (https://portal.gdc.cancer.gov/). Processed data and analysis scripts are available from the corresponding author upon reasonable request.
